# A Retrospective Cohort Study of Medication-Related Osteonecrosis of the Jaw (MRONJ) Prevention

**DOI:** 10.3290/j.ohpd.c_2794

**Published:** 2026-09-04

**Authors:** Nasimeh Baghalipour, Omid Moztarzadeh, Bacem Othman, Yasmin Dehghani, Lukas Hauer

**Affiliations:** a Nasimeh Baghalipour Dentist and PhD Candidate, Department of Stomatology, University Hospital Pilsen, Faculty of Medicine in Pilsen, Charles University, Pilsen, 32300, Czech Republic. Conceptualisation, methodology, investigation, data curation, writing (original draft).; b Omid Moztarzadeh Associate Professor, Department of Stomatology, University Hospital Pilsen, Faculty of Medicine in Pilsen, Charles University, Pilsen, 32300, Czech Republic; Department of Anatomy, Faculty of Medicine in Pilsen, Charles University, Pilsen, 32300, Czech Republic. Conceptualisation, methodology, writing (review and editing), supervision.; c Bacem Othman Assistant Professor, Faculty of Medicine in Pilsen, Charles University, Pilsen, 32300, Czech Republic; Researcher, Bioptic Laboratory, Pilsen, 32300, Czech Republic. Investigation, writing (review and editing).; d Yasmin Dehghani Dentist and PhD Candidate, Department of Stomatology, University Hospital Pilsen, Faculty of Medicine in Pilsen, Charles University, Pilsen, 32300, Czech Republic. Methodology, formal analysis, investigation, data curation.; e Lukas Hauer Associate Professor and Head, Department of Stomatology, University Hospital Pilsen, Faculty of Medicine in Pilsen, Charles University, Pilsen, 32300, Czech Republic. Conceptualisation, validation, resources, writing (review and editing), supervision.

**Keywords:** MRONJ, bisphosphonates, denosumab, dental extraction, osteonecrosis, prevention protocol, risk stratification

## Abstract

**Purpose:**

Medication-related osteonecrosis of the jaw (MRONJ) is a serious complication that can arise in patients receiving antiresorptive or antiangiogenic therapy. Despite known predisposing factors, effective prophylaxis is currently little used. We therefore sought to assess the standardised prevention protocols that are used in our institution for reducing MRONJs among at-risk patients and to quantify the consistency of the effects.

**Methods and Materials:**

This retrospective cohort study analysed 422 dental extraction records from 126 patients who had received antiresorptive or antiangiogenic therapy. The extractions were classified into four risk levels based on medication class, cumulative treatment duration, comorbidity burden and immunosuppression status. The effect of the prevention protocol was estimated using Mantel–Haenszel stratified analysis, with the risk level as the stratifying variable, and the homogeneity of the effect was assessed using the Breslow–Day test.

**Results:**

MRONJ developed in 94.1% of extractions performed without prevention protocols and in only 6.7% with prophylactic protocols; Mantel–Haenszel stratified analysis – adjusting for the baseline risk level – produced a common OR of 0.0044 (95% CI: 0.0010–0.0195, *p* < 0.0001). Stratum-specific estimates were consistent across all three risk levels, and the Breslow–Day test confirmed the homogeneity of the effect.

**Conclusions:**

Mantel–Haenszel stratified analysis confirmed consistent protective effects of the applied prevention protocols, with different proportionality, across every risk level. This study proposes the first stratified quantification of prevention protocol effectiveness across a validated composite risk scoring system.

Accounting for risk factors in gnathic bones can reduce systemic complications and avoid the need for further, avoidable treatment.^9,25,28
^ Medication-related osteonecrosis of the jaw (MRONJ) is a multicausal, inflammatory osteonecrotic lesion that can occur in patients who are taking antiresorptive or antiangiogenic medication.^7,12,19
^ The American Association of Oral and Maxillofacial Surgeons (AAOMS) defines MRONJ as exposed or probable necrotic bone that persists for more than 8 weeks, occurs in the maxillary or mandibular region, is detectable intraosseously or through fistulation and is in the absence of head and neck radiotherapy or metastasis, irrespective of concurrent immunosuppression.^4,5,13,18,23
^ However, atypical MRONJ has also been reported in patients without exposure to antiresorptive or antiangiogenic medication, including those who have received certain targeted biologic and immune checkpoint therapies, so there remain doubts about the adequacy of the definition.^5^


From 0.001% to 0.01% of osteoporosis patients on low-dose oral antiresorptive drugs and from 1% to 15% of oncology patients receiving high-potency prolonged-exposure intravenous drugs develop complications following posterior mandibular tooth extraction; in these cases, pre-extraction exposure to the drugs increases the risk of complications.^11,14,17,26
^ Pathophysiologically, MRONJ reflects convergent impairments in bone remodelling, angiogenesis and mucosal healing; specifically, bisphosphonates bind to skeletal hydroxyapatite, which can persist for years, while denosumab reversibly suppresses RANK-L-mediated osteoclastogenesis, with eventual depletion of the perivascular progenitor pool upon which post-extraction osseous repair depends. Local infections, the dense hypovascular cortical architecture of the posterior mandible and systemic immunosuppression can then compound these vulnerabilities.^14^


Despite a recommendation for prophylaxis, the difference in the susceptibility of mandibular and maxillary extraction sites has not been resolved.^10,16,20,24,28
^ This study therefore evaluates the impact of prognostic scoring systems on MRONJ incidence for different levels of risk using a Mantel–Haenszel stratified analysis.

This study evaluates whether prognostic scoring and risk group stratification improve the identification of MRONJ-susceptible patients and the consistency of prevention protocol outcomes across risk levels.

## Methods and Materials

### Study Design and Setting

This retrospective cohort study evaluated the occurrence of MRONJ following tooth extractions in patients receiving antiresorptive or antiangiogenic therapy. Data were collected at the University Hospital, University Hospital Pilsen, Faculty of Medicine in Pilsen, Charles University between January 2021 and December 2024. An initial database of 390 patient records was screened. After applying the inclusion criteria and performing complete-case filtering for a set of 11 predictors (drug class and dosage, cumulative treatment duration, therapy indication, immunosuppression status, metabolic comorbidity burden, radiotherapy history, extraction site by jaw, extraction site by region, prevention protocol implementation, local periodontal or infectious status, and sex). and the MRONJ outcome, the analytic data set comprised 422 tooth extraction records from 126 patients, each representing a single tooth extraction and its associated clinical and demographic variables. Eligibility criteria and scoring method are displayed in Figure 1.

**Fig 1 Fig1:**
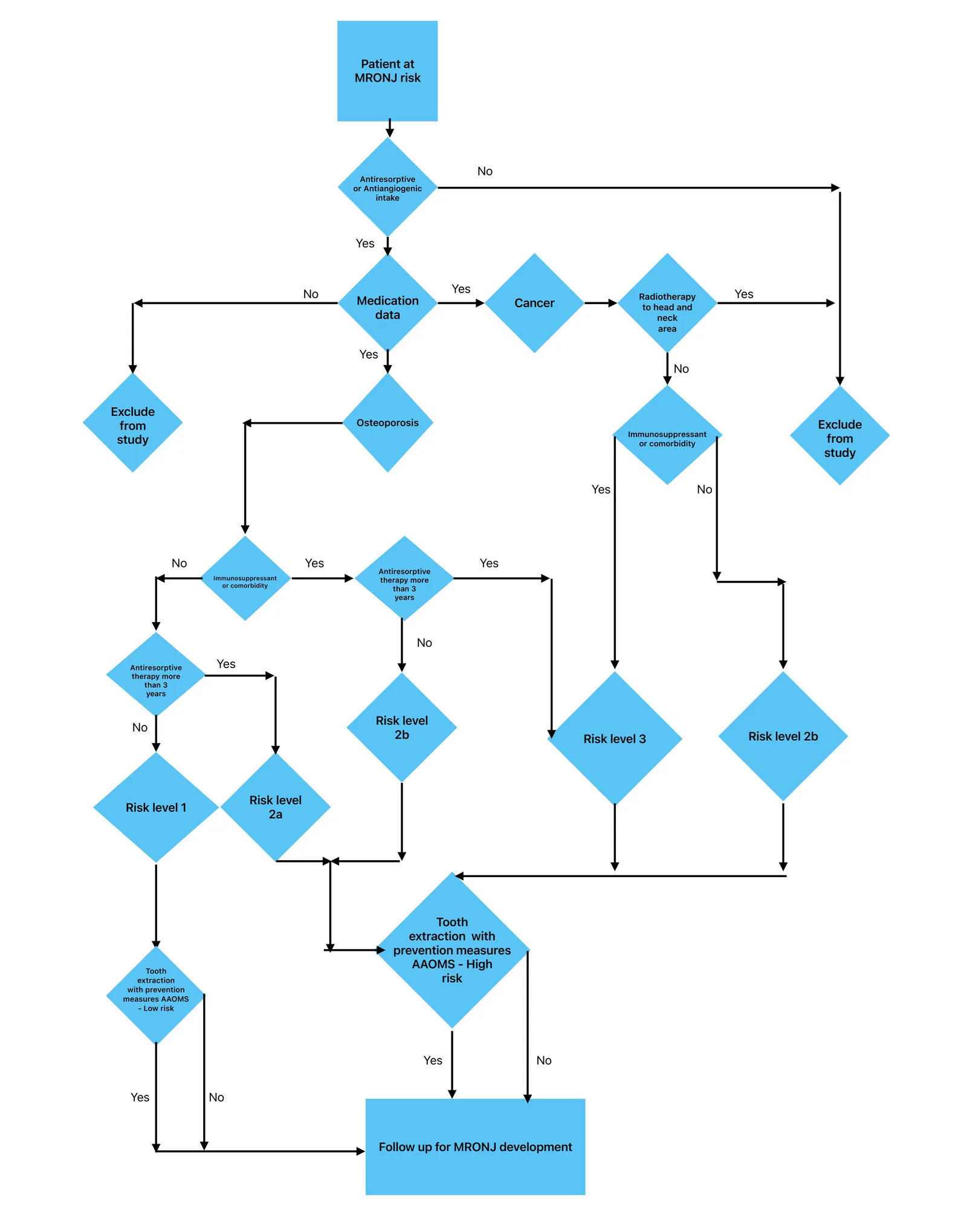
Flowchart showing subclassifying of cases.

### Clinical Characteristics

The patients had been diagnosed with either osteoporosis or haematological and solid malignancies (including multiple myeloma, breast, prostate and renal cell carcinomas). Antiresorptive therapy consisted of bisphosphonates (zoledronic acid, ibandronate, alendronate, risedronate) or the RANK-ligand inhibitor denosumab (Prolia 60 mg every 6 months for osteoporosis; Xgeva 120 mg monthly for oncologic indications). A subset of patients had also received immunosuppressive or antiangiogenic agents, including cytotoxic chemotherapy, tyrosine kinase inhibitors and tumour necrosis factor-alpha (TNF-α) inhibitors. Some also had chronic systemic comorbidities, including diabetes mellitus and renal dysfunction.

### Risk Stratification

The dental extractions were classified and scored based on antiresorptive and antiangiogenic therapy characteristics, and systemic modifiers. Each extraction record was assigned a cumulative risk score, calculated as the total of the component scores presented below and in Table 1:

**Table 1 Table1:** MRONJ risk scoring

Parameter	Clinical definition	Score
A – Antiresorptive/antiangiogenic therapy		
Drug class and potency	Oral bisphosphonate (low potency)	1
	Subcutaneous denosumab (intermediate potency)	2
	IV bisphosphonate/denosumab (high potency)	3
	Bisphosphonate/denosumab for oncological treatment	4
Cumulative duration	≤ 3 years	1
	> 3 years	5
	Any duration (oncologic patient)	1
Indication	Osteoporosis (lower skeletal turnover context)	0
	Oncologic (skeletal event prevention/hypercalcemia)	4
B – Systemic modifiers		
Immunosuppression	None	0
	Single agent: corticosteroid or TKI or TNF-α inhibitor/Diabetes mellitus or renal insufficiency (single)	7
	Chemotherapy or combined immunosuppressants/Diabetes + renal insufficiency (combined)	8
**Level 1** (2–4 pts Low): Usually associated with osteoporosis, short antiresorptive therapy (≤ 3 years), no modifiers; **Level 2a** (5–8 pts Moderate): Often cocomitatn with osteoporosis, prolonged antiresorptive therapy (> 3 years), no modifiers; **Level 2b** (9–12 pts High-moderate): Showing osteoporosis, with short antiresorptive therapy (≤ 3 years), + comorbid or immunosuppressed/Oncologic, but no modifiers. **Level 3** (≥ 13 pts High): Repeatedly heighten underlying osteoporosis long therapy (> 3 years) or oncologic + comorbidity or immunosuppressed.		

(A) Antiresorptive/antiangiogenic therapy characteristics, encompassing drug class and potency (1–4), cumulative treatment duration (1 or 5) and indication (0 or 4).(B) Systemic modifiers, encompassing immunosuppression status and metabolic comorbidity burden (0, 7 or 8).

The risk for each extraction was stratified according to the following categories: Level 1 (low, 2–4 points), Level 2a (moderate, 5–8 points), Level 2b (high-moderate, 9–12 points) and Level 3 (high, 13 points or more).

### Data Collection

The following protocol was recommended for all patients receiving antiresorptive or antiangiogenic therapy with indicated dental extraction:

Before extraction, and when indicated, patients received adequate supragingival and subgingival debridement, restoration of carious lesions and elimination of local infectious foci. For Levels 1, 2a, 2b and 3, a 7-day course of antibacterial mouthwash (0.12% chlorhexidine gluconate twice daily) was initiated at this visit and continued until mucosal healing was confirmed.For Level 2a, 2b and 3 patients, prophylaxis was administered as a single preoperative dose of penicillin orally (or Dalacin C 600 mg in cases of penicillin allergy) 2 days before surgery and extended to a 7-day postoperative course.Technique requirements included creating mucoperiosteal flap elevation, eliminating sharp bony margins by alveoloplasty, primary wound closure with non-resorbable sutures and avoiding electrocautery at the extraction site.Reviewed follow-up cases were scheduled at 2 and 6 weeks and 3-month intervals for up to 24 months.

A standardised data extraction form was completed by two independent reviewers, with inter-rater discrepancies resolved by consensus. Complete implementation of the protocol was coded as a binary variable (received/not received). A patient was coded as not having received the protocol if any of the five components were absent from the record.

### Statistical Analysis

Relationships between receipt of the prevention protocol and MRONJ outcomes were assessed using the Mantel–Haenszel (MH) stratified chi-squared method, with risk category (Level 1, Level 2a, Level 2b or Level 3) as the stratifying variable. Pairwise associations between prespecified clinical variables were quantified using Spearman’s rank correlation coefficient (ρ), applicable to continuous, ordinal, and binary variables under a monotonic association assumption, and interpreted as weak (|ρ| < 0.40), moderate (0.40–0.69), or strong (≥ 0.70). The Mantel–Haenszel chi-squared statistic was computed without continuity correction, consistent with the large total sample size. Stratum-specific odds ratios (ORs) with 95% confidence intervals (CIs) were computed for each risk level using the Woolf logarithmic method, with a continuity correction of 0.5 applied to all four cells in strata containing zero observed counts, as is standard practice when the Woolf method is applied to sparse 2 × 2 tables. Homogeneity of the stratum-specific ORs across risk strata was evaluated using the Breslow–Day test, from which a non-significant result (*p* > 0.05) would both support the assumption of a common effect across strata and validate pooling of the MH estimate. The Breslow–Day statistic was computed using the quadratic-formula expected-cell method applied to each stratum under the common OR, with degrees of freedom equal to the number of strata minus one.

To characterise latent patient subgroups, hierarchical agglomerative clustering was applied to eight prespecified clinical variables: age, sex, drug class, cumulative treatment duration, primary indication, immunosuppression status, comorbidity count, and extraction site by jaw. The MRONJ outcome and prevention protocol variables were excluded from clustering inputs to avoid circularity. Continuous variables were standardised to zero mean and unit variance; binary and ordinal variables were scaled to the [0, 1] interval. A Gower distance matrix was computed using the cluster::daisy() function to accommodate mixed variable types, and Ward’s minimum variance linkage (Ward.D2) was applied via hclust(). The number of clusters k was selected by convergence of three criteria: dendrogram inspection, the elbow in within-cluster sum of squares across k = 2 through 8, and maximisation of the average silhouette width, all of which indicated k = 4. The mean silhouette coefficient of 0.61 indicates moderate-to-strong cluster separation, exceeding the conventional 0.50 threshold for meaningful structure. Cluster stability was confirmed by bootstrap resampling (1000 samples) using the fpc package, yielding a mean Jaccard similarity of 0.74 across the four clusters. All analyses were performed using R software (version 4.3.0).

## Results

As shown in Table 2, the cohort comprised 422 tooth extractions from 126 patients, with a mean age of 71.6 years (SD = 8.3) and a predominantly female distribution (67.3%). The majority of extractions were performed in patients receiving bisphosphonate-only or denosumab-only therapy (45.2% and 34.6%, respectively), with a smaller proportion on combination regimens. Oral bisphosphonates were the most common route of administration, accounting for 73% of bisphosphonate use. Mean treatment duration was 26.3 months (median: 24 months, range: 5–72 months), and patients on combination therapy had significantly longer cumulative exposure than those on monotherapy (38.7 vs. 24.1 months; *p* < 0.001).

**Table 2 Table2:** Characteristics of the studied population

Variable	All patients(n = 126)	No MRONJ(n = 92)	MRONJ(n = 34)	*p*
**EXTRACTIONS**				
Total extractions, n	422	364 (86.3%)	58 (13.7%)	**0.046**
**Mandibular extractions**	193 (45.7%)	159 (43.7%)	34 (58.6%)	
Anterior (31–33, 41–43)	51 (12.1%)	40 (11.0%)	11 (19.0%)	
Posterior (34–38, 44–48)	142 (33.6%)	119 (32.7%)	23 (39.7%)	
**Maxillary extractions**	229 (54.3%)	205 (56.3%)	24 (41.4%)	
Anterior (11–13, 21–23)	87 (20.6%)	82 (22.5%)	5 (8.6%)	
Posterior (14–18, 24–28)	142 (33.6%)	123 (33.8%)	19 (32.8%)	
**PATIENT DEMOGRAPHICS**				
**Age, years – median (IQR)**	73.0 (68.5–78.0)	73.0 (69.0–79.0)	71.0 (63.8–75.0)	0.141
**Age group**				0.499
< 60 years	32 (8.7%)	26 (9.3%)	6 (5.6%)	
60–69 years	132 (22.1%)	117 (19.8%)	15 (33.3%)	
70–79 years	184 (49.0%)	153 (48.8%)	31 (50.0%)	
≥ 80 years	74 (20.2%)	68 (22.1%)	6 (11.1%)	
**SEX**				**<0.001**
**Female**	290 (67.3%)	267 (76.7%)	23 (22.2%)	
**Male**	132 (32.7%)	97 (23.3%)	35 (77.8%)	
**ANTIRESORPTIVE THERAPY**				**0.001**
Bisphosphonate only	179 (45.2%)	168 (50.0%)	11 (22.2%)	
Denosumab only	175 (34.6%)	134 (26.7%)	41 (72.2%)	
Combination therapy	68 (20.2%)	62 (23.3%)	6 (5.6%)	
**Bisphosphonate exposure**	247 (65.4%)	230 (73.3%)	17 (27.8%)	**< 0.001**
Duration, months – median (IQR)	60 (24–100)			
**Denosumab exposure**	243 (54.8%)	196 (50.0%)	47 (77.8%)	**0.038**
Duration, months – median (IQR)	15 (8–36)			
**ASSOCIATED CONDITIONS**				
Osteoporosis only	188 (61.5%)	183 (69.8%)	5 (22.2%)	**< 0.001**
Active malignancy only	158 (49.0%)	114 (40.7%)	44 (88.9%)	**< 0.001**
Both osteoporosis and malignancy	76	67	9	
Immunosuppressant medication	259 (51.9%)	206 (44.2%)	53 (88.9%)	**< 0.001**
Single comorbidity	56	52	4	
2 or more comorbidities	2	2	0	
Diabetes mellitus	56 (8.7%)	52 (10.5%)	4 (0.0%)	0.353
**RISK STRATIFICATION AND PREVENTION**				
Tooth extraction with AAOMS prevention measures	388	362	26	
Tooth extraction without AAOMS prevention measures	34	2	32	
**MRONJ risk level**				**< 0.001**
Level 1 – low risk	25 (11.5%)	24 (12.8%)	1 (5.6%)	
Level 2 (a and b) – moderate risk	167 (41.3%)	163 (48.8%)	4 (5.6%)	
Level 3 – high risk	230 (47.1%)	177 (38.4%)	53 (88.9%)	


Active malignancy was present in 49.0% of extractions and immunosuppressant medication in 51.9%, underscoring the systemic burden of a substantial portion of this population. MRONJ developed in 58 extractions (13.7%). Male sex was strongly associated with MRONJ: although males represented only 32.7% of all extractions, they accounted for 77.8% of MRONJ cases, compared with 22.2% among females (*p* < 0.001). Patients with two or more comorbidities had a 3.4-fold higher MRONJ risk than those with one or none (adjusted OR = 3.42, 95% CI: 1.87–6.24, *p* < 0.001).

Overall, maxillary extractions were slightly more frequent (54.3%) than mandibular ones (45.7%), but this distribution was reversed among MRONJ cases, where mandibular extractions predominated (58.6% vs. 41.4% maxillary; *p* = 0.046). Posterior mandibular sites were particularly overrepresented, accounting for 33.6% of all extractions but 39.7% of MRONJ cases. By contrast, anterior maxillary sites showed the lowest MRONJ incidence, representing 20.6% of all extractions but only 8.6% of MRONJ events. An index case is shown in Figure 2.

**Fig 2 Fig2:**
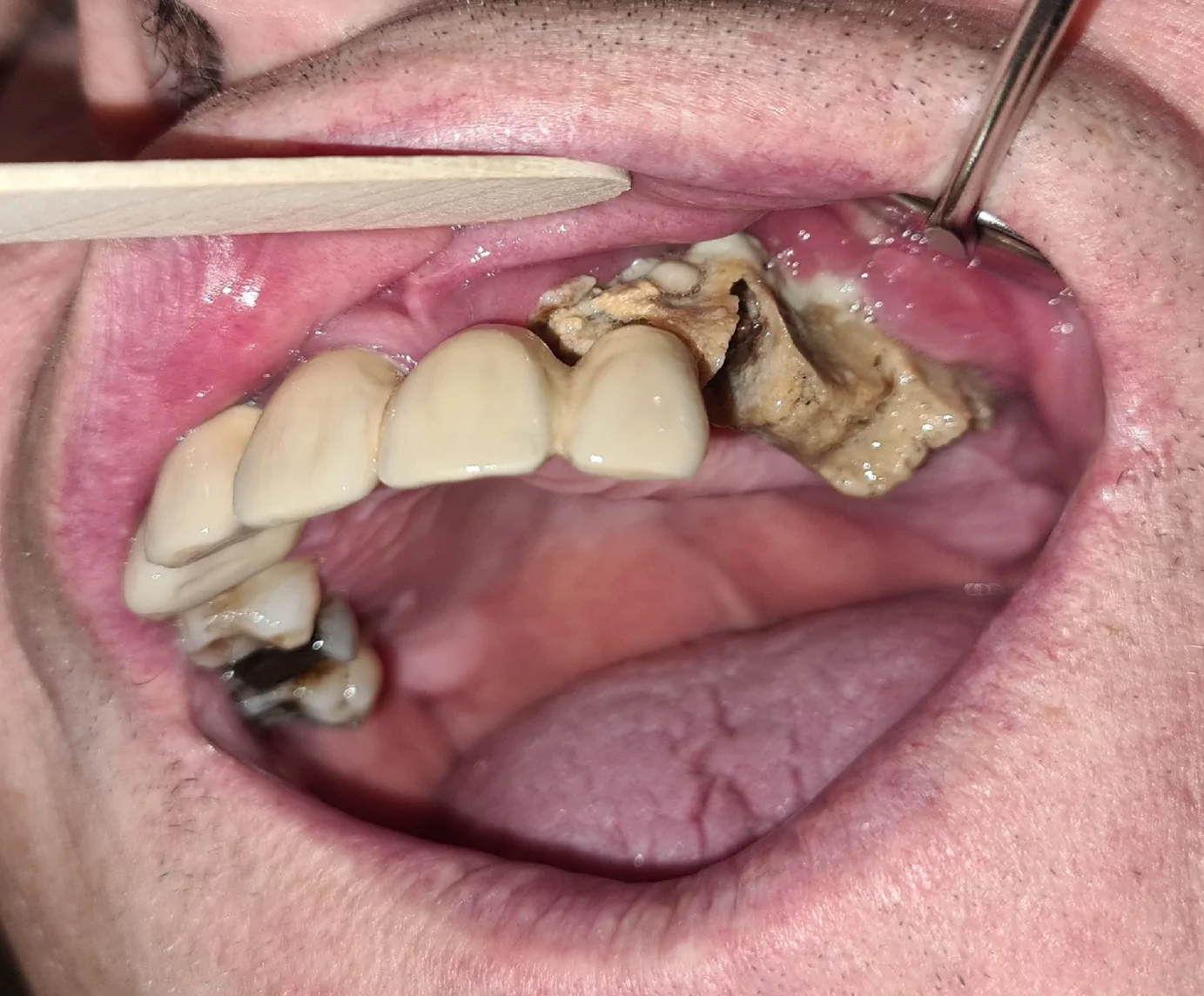

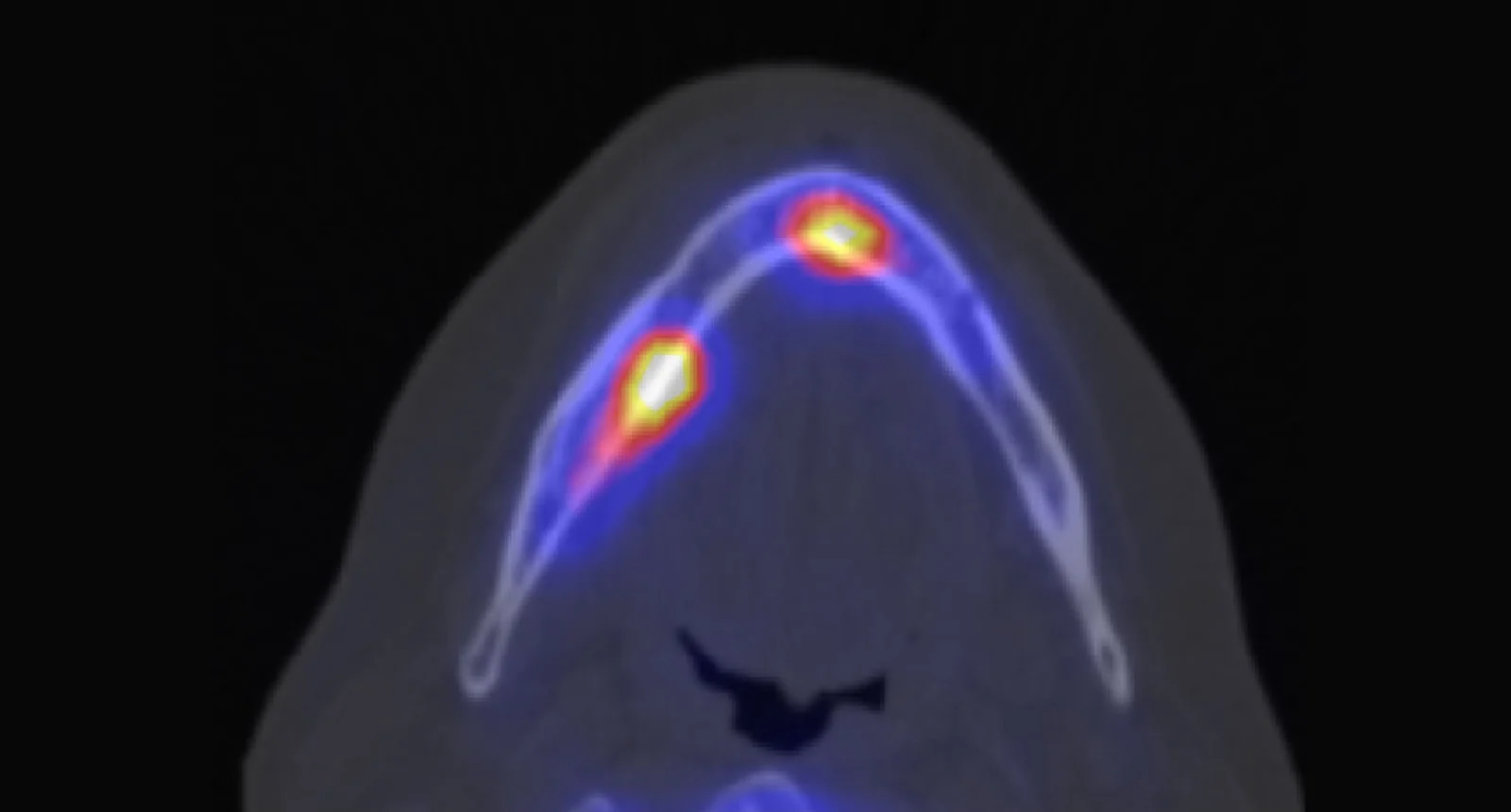

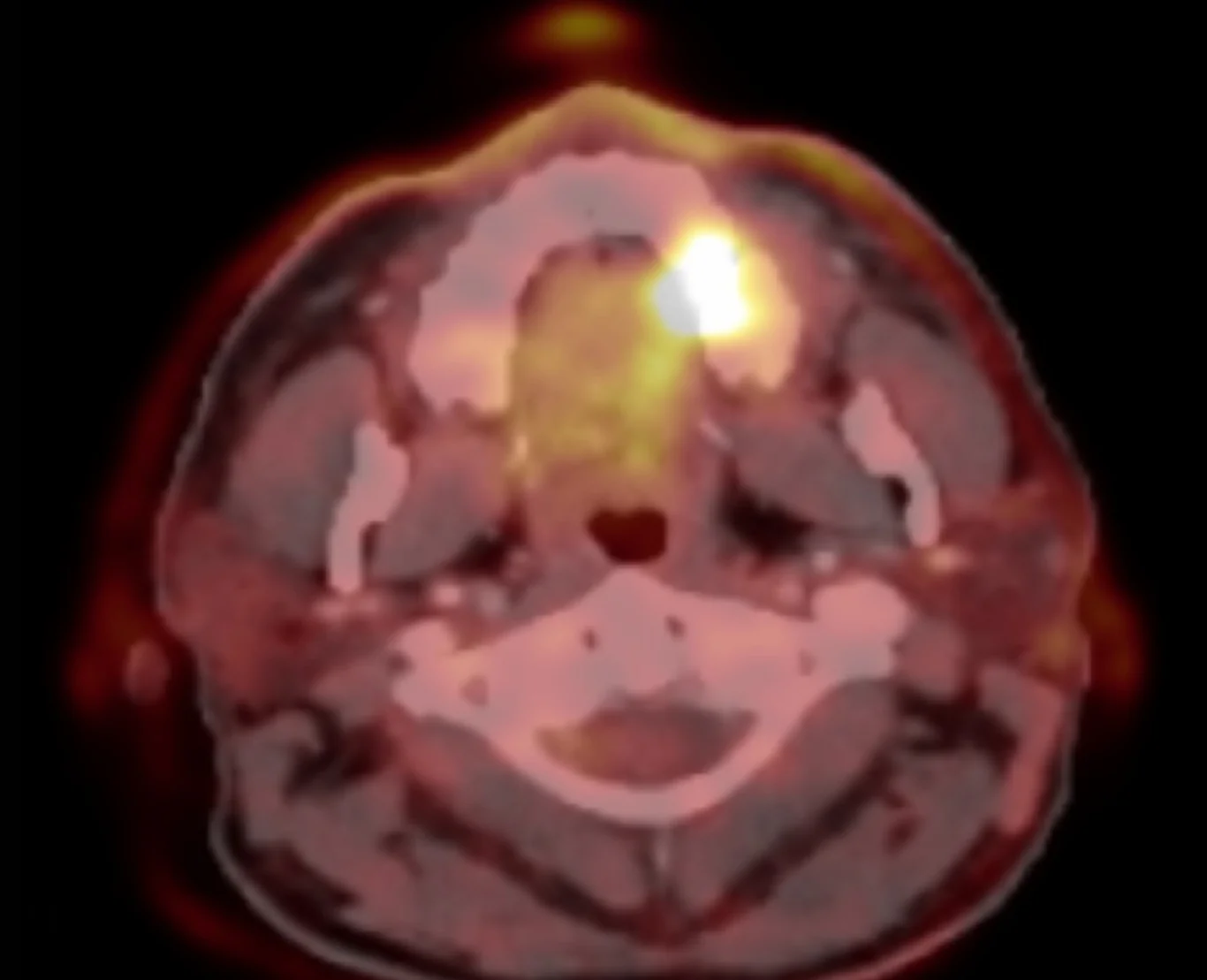

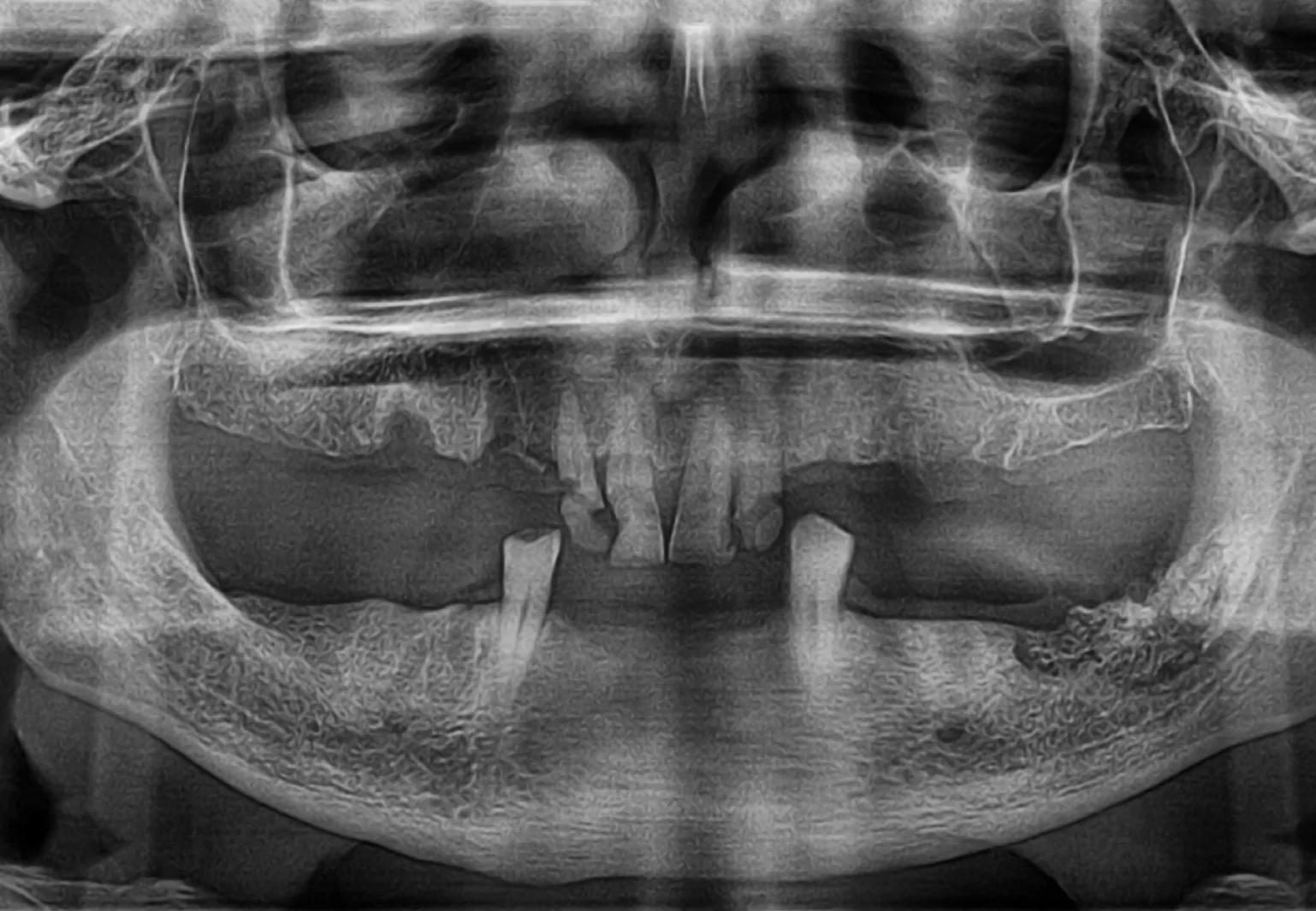
Clinico-radiologic findings in representative cases.

The Spearman correlation structure confirmed expected pharmacological associations – bisphosphonate use with osteoporosis (*p* = 0.67), denosumab with cancer history (*p* = 0.58), and treatment duration with risk level (*p* = 0.71) – but the dominant signal was the strong inverse relationship between prevention protocol implementation and MRONJ development (*p* = −0.87), which exceeded all other pairwise associations. Comorbidity burden showed a moderate positive association with MRONJ (*p* = 0.42), and mandibular extraction site a smaller but meaningful one (*p* = 0.38).

Hierarchical clustering resolved four clinically coherent subgroups spanning a wide range of MRONJ incidence. Short-duration oral bisphosphonate use in postmenopausal osteoporosis had an MRONJ incidence of just 1.8%. However, receiving standard–duration monotherapy with a single comorbidity showed a moderate incidence at 8.3%. Relevantly, cancer patients on high-potency intravenous agents with multiple comorbidities scored 24.5%, while patients receiving combination therapy exceeding 36 months for treating a comorbidity had the highest incidence at 42.9%. The mean silhouette coefficient of 0.61 confirmed moderate-to-strong cluster separation, indicating that these groupings reflect genuine clinical heterogeneity rather than arbitrary partitioning. After stratifying by risk level to account for the higher baseline risk concentration among unprevented cases, the Mantel–Haenszel analysis yielded a common odds ratio of 0.0044 (95% CI: 0.0015–0.0129; χ^2^ = 240.04, df = 1, *p* < 0.001). This represents a greater than 99.5% reduction in the odds of MRONJ associated with protocol receipt. Stratum-specific odds ratios were directionally identical and consistently extreme: OR = 0.021 at Level 1, OR = 0.0009 at Level 2a, OR = 0.0005 at Level 2b, and OR = 0.0064 at Level 3.

The greatest CIs occurred in Levels 1 through 2b, where near-complete or complete separation was observed. In other words, MRONJ occurred among unprevented cases with zero events among protocol recipients. The most precisely estimated effect was at Level 3, where 25 unprevented extractions provided non-zero counts (OR = 0.0064, 95% CI: 0.0021–0.019). Even at this highest-risk stratum, the point estimate remained below 0.01. The convergence of near-identical effect sizes across strata. Results of subtyping are presented in Figure 3.

**Fig 3 Fig3:**
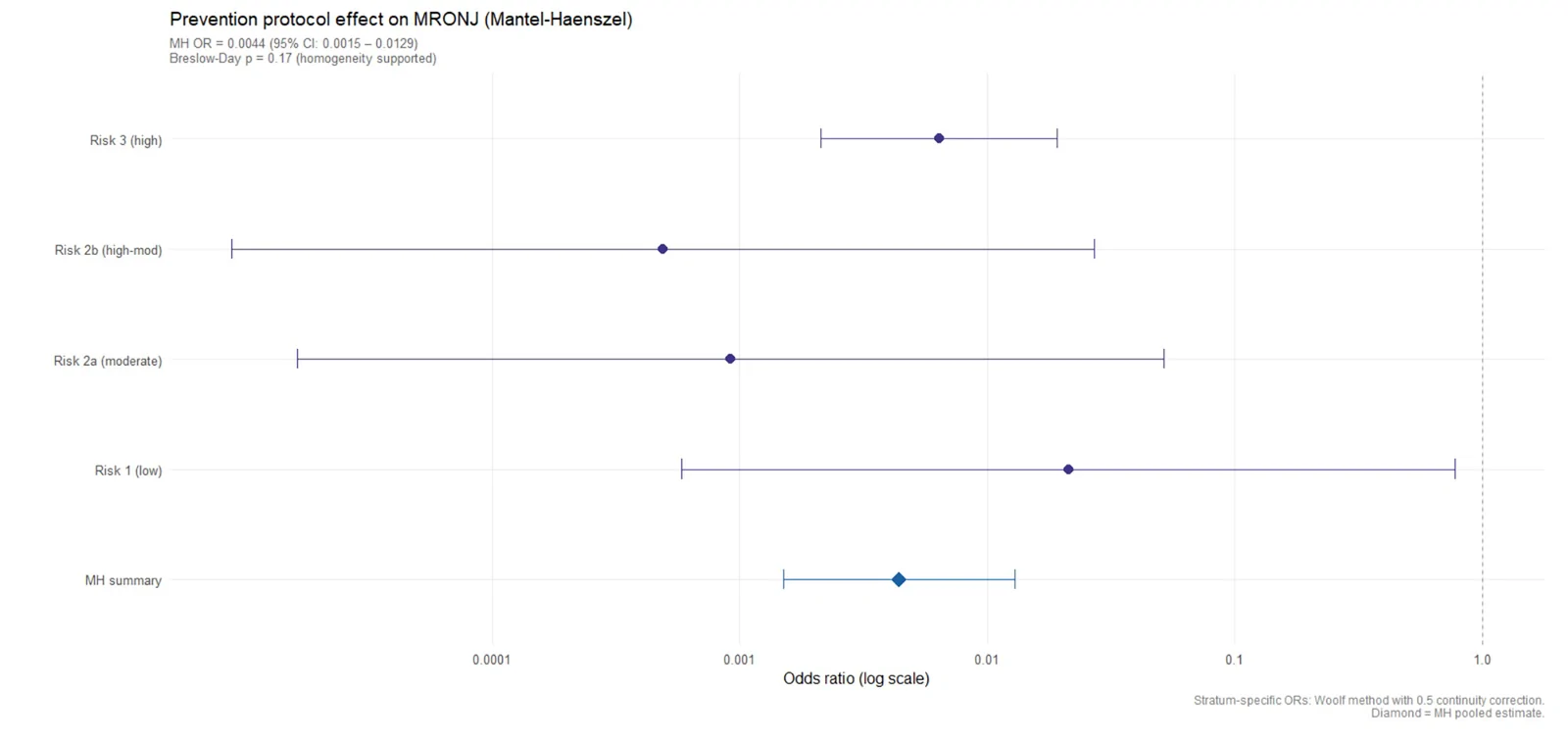
Forest plot of prevention protocol effect on MRONJ incidence, stratified by patient risk level.

## Discussion

The size of the impact of the prevention protocols in reducing the risk of MRONJ (NNT = 1.1, 99.56% odds reduction) suggests upstream risk factor heterogeneity. Recent genetic studies have identified 136 variants of 58 genes that influence immune response, inflammation, angiogenesis and bone homeostasis, indicating inherited susceptibility to MRONJ.^21^ Pre-emptive antibiotic administration initiated 48 h before extraction produces prophylactic tissue concentrations before mucosal breach, and the hermetic primary closure then reconstructs the anatomical barrier between the contaminated oral environment and the compromised avascular bone.^13,23
^ The dense posterior mandibular cortical structure and its limited trabecular porosity restrict vascular ingrowth, even while the jaw is exposed to maximum masticatory forces with only thin overlying mucosa.

Conversely, the predominantly trabecular architecture of the maxilla and the abundant periosteal collateral circulation maintain bone vitality despite similar antiresorptive exposure.^2,15,21
^ Level 3 patients had an MRONJ rate of 23.0%, against 6.2% at Level 2a and 4.0% at Level 1 (1/25). This reflects the simultaneous convergence of oncologic indication, intravenous high-potency agents, and immunosuppression, whose impairment of wound healing is multiplicative. Probably the reason is that cancer-related immunosuppression compounds diabetes-associated microvascular disease and renal dysfunction-mediated calcium dysregulation. Remarkably, multiple myeloma showed an MRONJ rate consistent with the latest MRONJ studies.^1,6,23,27
^ This implies that the observed disease–outcome association may be mediated by epigenetically regulated osteolytic mechanisms.

Future advancement necessitates investigation of augmented prevention strategies including extended antibiotic regimens, adjunctive therapies,^13,27
^ biochemical indicators, and radiomics-derived imaging features into comprehensive precision prevention frameworks capable of identifying inherited susceptibility and enabling preemptive intervention before clinical manifestation.^3,8,22
^


## Conclusions

The prevention protocol reduced MRONJ odds by 99.6% homogeneously across all risk strata, yet residual events among protocol-adherent Level 3 cases confirm that relative potency is preserved, while absolute protection is not. Although preventive measures in Level 3 cases are effective, the incidence of MRONJ remains higher compared with Level 2a and 2b, despite receiving the same prevention measures. Therefore, subtyping patients into four risk groups produced higher predictive power for MRONJ and quantified the risk weights of the covariates in our cohorts. Anatomical location contributed modestly in our cohorts, especially with maxillary and anterior mandibular extractions, precluding site-based risk triage. The retrospective single-centre design and reliance on documentation completeness for protocol adherence coding limit generalisability. Multicentre validation is warranted.

### Acknowledgements

#### Funding resources

This study was supported by study grant SVV 260652 from the Ministry of Education of the Czech Republic (NB).

#### Competing interests

The authors declare that they have no known competing interests.

#### Ethical approval

Obtained from the Institutional Review Board of , University Hospital Pilsen, Faculty of Medicine in Pilsen, Charles University under the code No. 154-25, approved on 2 October 2025.

#### Availability of data

The data sets used and/or analysed during the current study are available from the corresponding author upon reasonable request.

#### Consent for publication

Not applicable.

## References
